# Use of Jianpi Jiedu Herbs in Patients with Advanced Colorectal Cancer: A Systematic Review and Meta-Analysis

**DOI:** 10.1155/2018/6180810

**Published:** 2018-01-29

**Authors:** Shaofan Zhang, Li Shi, Dan Mao, Weijun Peng, Chenxia Sheng, Chenchen Ding, Fengxia Lin, Caiyun Lei, Sifang Zhang

**Affiliations:** ^1^Department of Integrated Chinese and Western Medicine, The Second Hospital of Xiangya, Central South University, Changsha 410008, China; ^2^Department of Cardiology, Affiliated Bao'an TCM Hospital, Guangzhou University of Traditional Chinese Medicine, Shenzhen, Guangdong, China

## Abstract

**Objective:**

To systematically review the effect of invigorating Pi and detoxification (Jianpi Jiedu, (JPJD)) herbs in advanced colorectal cancer (CRC) patients receiving chemotherapy.

**Methods:**

Three English and four Chinese databases were searched. Literature was screened by EndNote X7 and data were analyzed by RevMan 5.2.

**Results:**

This review comprised 12 randomized clinical studies of 701 patients. The results showed that JPJD herbs improved the therapeutic effect on Chinese medicine symptoms [risk ratio (RR) = 1.59; 95% confidence interval (CI): 1.35~1.88] and Karnofsky performance score [RR = 2.07; 95% CI: 1.52~2.82] for advanced CRC patients receiving chemotherapy, lowered the Chinese medicine symptoms' score [weighted mean difference = −2.44; 95% CI: −3.23~−1.64], reduced the incidence of nausea and vomiting [RR = 0.23; 95% CI: 0.11~0.49], improved platelet at toxicity grades III-IV [odds ratio = 0.29; 95% CI: 0.12~0.74] and I–IV [RR = 0.65; 95% CI: 0.51~0.82], and improved white blood cell at toxicity grades III-IV [RR = 0.37; 95% CI: 0.23~0.58] and I–IV [RR = 0.69; 95% CI: 0.60~0.79]. However, the results showed no significant effect on tumor response.

**Conclusion:**

JPJD herbs can improve quality of life, relieve symptoms, and reduce adverse events of advanced CRC patients receiving chemotherapy.

## 1. Introduction

Colorectal cancer (CRC), including colon and rectal cancer, is typically a malignancy of the intestinal epithelial mucosa and is one of the most common gastrointestinal malignancies. CRC is the third most common type of malignant tumor in China [1]. Worldwide, after lung and breast cancer, CRC is the most prominent cancer impacting disability-adjusted life years (DALY) [2]. According to the International Agency for Research on Cancer (IARC), in 2012, there were estimated to be 1.36 million new cases and 0.69 million deaths from CRC worldwide [3]. Currently, CRC treatment options include surgery, chemotherapy, radiotherapy, or a combination of the three [4, 5]. Surgery is usually the first option, with the excision rate reaching 60–70% [6]. However, more than half of these patients relapse or present with metastases in 5 years, ultimately succumbing to the disease. In China, CRC is most prevalent in those aged 40–60 years. Due to its occult onset and poor patient awareness of the disease, most are diagnosed at an advanced stage [7]. In such cases, it is usually too late for surgery, with chemotherapy being the main form of adjuvant therapy [8]. Chemotherapy can prolong survival and a randomized crossover trial has shown that patients receiving chemotherapy, in any sequence, survive a median period of over 20 months [9]. Unfortunately, chemotherapy of CRC is often ineffective due to the intrinsic chemoresistance of the tumor [10]. Overall CRC survival rates are unsatisfactory, especially for late-stage disease [11]. Hence, it is imperative to develop more effective treatments for CRC.

Multiple studies [12–14] have demonstrated traditional Chinese medicine (TCM) to enhance efficacy and to decrease the toxicity of chemotherapy. TCM has been shown to alleviate symptoms, improve quality of life, enhance immune function, and relieve the adverse effects of chemotherapy [15]. According to TCM, tumor formation results from the accumulation of toxicants from Zang-Fu. The rise and fall of healthy qi is the key factor, playing an important role in the occurrence, development, and turnover of disease. In CRC, a deficiency of healthy qi is mainly due to spleen deficiency and due to pathogenic factors associated with toxic heat. Spleen deficiency is the root cause and toxic heat is the manifestation, and each of these occurs during the development and progression of CRC. Hence, invigorating the spleen and detoxification (Jianpi Jiedu, (JPJD)) are the basic therapeutic methods of treatment for advanced CRC [16, 17].

In order to objectively assess the clinical efficacy of JPJD for the treatment of advanced CRC, this investigation assessed randomized controlled trials (RCTs) that used JPJD methods and herbs.

## 2. Methods

### 2.1. Database and Search Strategy

A comprehensive literature review was conducted on RCTs that combined treatments (JPJD herbs plus chemotherapy) for advanced CRC. Various databases were searched from the inception to March 2017, including the Cochrane Library, the Excerpta Medica Database (EMBASE), the Chinese Biomedical Literature Database (CBM), Wanfang Data (for unpublished graduate theses in China), China National Knowledge Infrastructure (CNKI), Chinese Scientific Journals Database (VIP), and PubMed/MEDLINE. The basic terms used were the following: (“Colonic Neoplasms” OR “Colonic Neoplasm” OR “Colon Neoplasms” OR “Colon Neoplasm” OR “Cancer of Colon” OR “Colon Cancers” OR “Cancer of the Colon” OR “Colonic Cancer” OR “Colorectal cancer” OR “Colonic Cancers” OR “colon cancer”) AND (“Chinese medicine” OR “Chinese herbal medicine” OR “Jianpi” OR “Jiedu”). Two reviewers (Shaofan Zhang and Li Shi) separately searched databases based on the same strategy. All identified literatures were screened after duplicate checking with EndNote X7.

### 2.2. Inclusion Criteria

The included studies met the following criteria: (1) randomized controlled trial (RCT); (2) diagnosis confirmed as advanced CRC, according to “New Criteria for the Diagnosis and Treatment of Common Malignant Tumors” [18], “Clinical Guidelines for new drugs in Traditional Chinese medicine” [19], and “Internal Medicine of Traditional Chinese Medicine” [20]; (3) intervention by administration of a Chinese medicine (CM) decoction with JPJD herbs and chemotherapy to patients (the treatment group), compared to the administration of chemotherapy alone (the control group), where the specific chemotherapy regimen in each study was not limited; (4) outcomes measures being as follows: CM symptoms score, therapeutic effect of CM symptoms, performance status, adverse events (AEs) of chemotherapy, and tumor response.

### 2.3. Exclusion Criteria

The excluded studies were excluded due to the following reasons: (1) studies did not meet the above criteria; (2) Chinese herbal medicine (CHM) was used in both the intervention group and the control group; (3) the study was not an original research (e.g., review article and letter to the editor); (4) studies were duplicated publications or articles without specific data; (5) there were no specific criteria for inclusion or exclusion; (6) they were laboratory studies.

### 2.4. Quality Assessment and Data Extraction

The process of literature screening and data extraction (using Excel) was carried out by two independent reviewers (Shaofan Zhang and Li Shi). Selection bias (random sequence generation and allocation concealment), performance bias (blinding of participants and personnel), detection bias (blinding of outcome assessment), attrition bias (incomplete outcome data), reporting bias (selective reporting), and other biases (determined according to sample size calculation method, inclusion/exclusion of criteria for patient's recruitment, comparability of baseline data, funding sources, and any other potential methodological flaw that might have influenced the overall assessment) were evaluated according to the criteria of the Cochrane Handbook for Systematic Reviews of Interventions [21]. Three potential bias judgments, low risk, high risk, and unclear risk, were determined for each trial. A judgment of low risk was made when all the items met the criteria of “low risk,” and a judgment of high risk for bias was made when at least one of the items was assessed as “high risk” [22, 23]. All risks for biased data are presented in Figures 2 and 3. Disagreements between the two reviewers were resolved by discussion with a third reviewer (Dan Mao).

### 2.5. Statistical Analysis

EndNote X7 (Thomson Reuters, USA) was used to manage the articles and the Cochrane Collaboration software (RevMan 5.2) was used to perform the statistical analysis. Weighted mean differences (WMD) with 95% confidence interval (CI) were calculated for continuous data, and standardized WMD (SMD) was calculated for data measured in different ways by each trial. Dichotomous data were expressed as relative risk (RR) or odds ratio (OR) with 95% confidence interval (CI). *χ*^2^ test and *I*^2^ test were used to assess the heterogeneity of the data. If heterogeneity existed in pooled studies (*I*^2^ ≥ 50%), a random model was applied; if not, a fixed model was applied. Statistically significant difference was considered as *p* < 0.05.

## 3. Results

### 3.1. Search Results and Study Characteristics

In this study, 1221 citations were identified through the electronic databases (the Cochrane Library, EMBASE, CBM, WF, CNKI, VIP, and PubMed/MEDLINE). Of these, 142 were rejected following duplicate checking with EndNote X7. Potentially relevant articles (*n* = 156) were retrieved through title and abstract checking. Each of these was assessed in detail. Articles relevant to “Jianpi” or “Jiedu” (*n* = 75) remained after further screening. Of these, 63 full-text articles did not fully meet inclusion criteria or were duplicate publications. Finally, only 12 studies [24–35] fulfilled the eligibility criteria.

**Figure 1:**
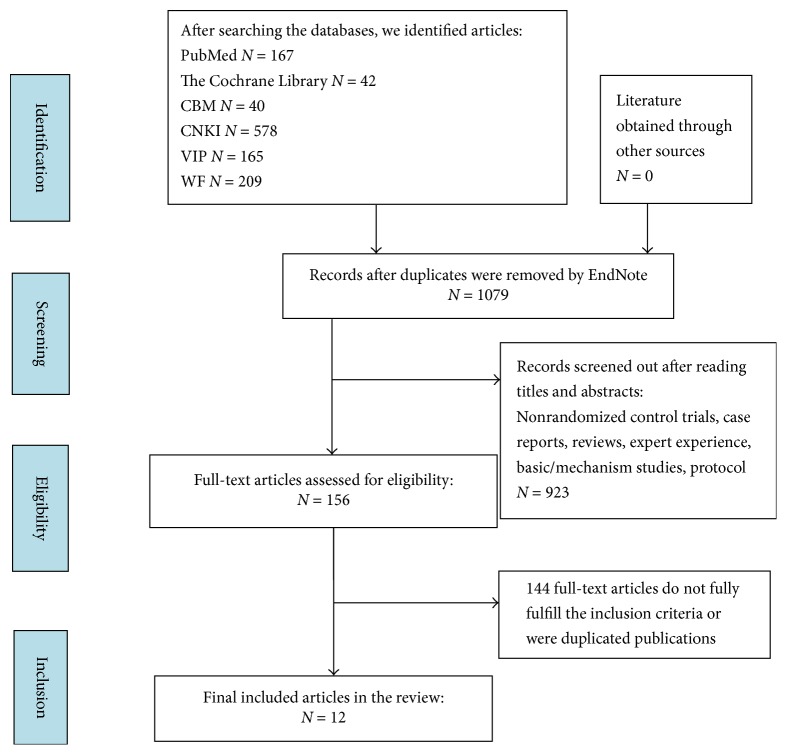
Flow diagram of included studies in the meta-analysis (up to March 2017).

A total of 701 patients were enrolled in the studies, of which 355 patients participated in chemotherapy combined with JPJD herbs (CTJ) and 346 received chemotherapy alone.  Figure 1 illustrates the process of literature screening and Table 1 shows the main characteristics of each trial included in the meta-analysis.

### 3.2. Risk of Bias

**Figure 2:**
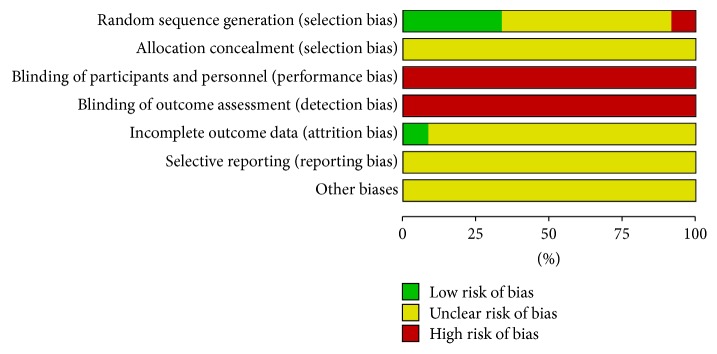
Risk of bias graph: review authors' judgements about each risk of bias item presented as percentages across all included studies.

**Figure 3:**
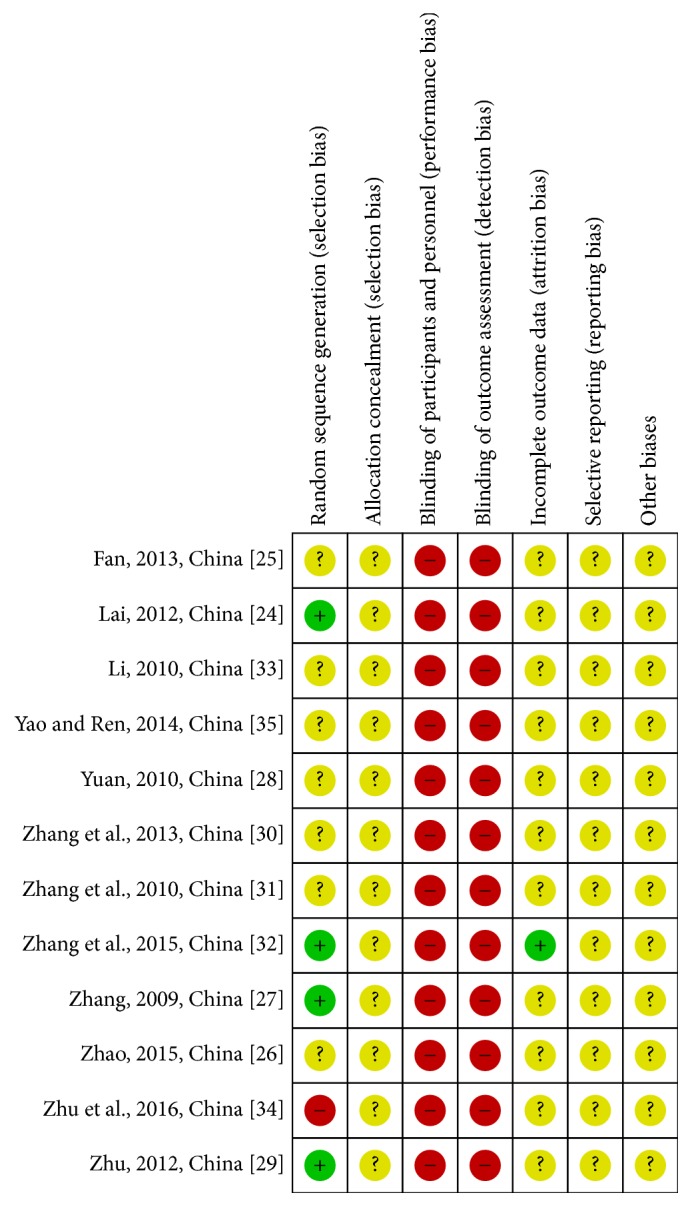
Risk of bias summary: review authors' judgements about each risk of bias item for each included study.

In general, the risk of bias in all 12 included articles was unclear or high in that limited information was available to the reviewers. Twelve studies mentioned randomization, but only 4 trials [24, 27, 29, 32] included a detailed description of the randomization method. These were considered as low risk and were divided into groups by random order. No study showed allocation concealment. Attempts to contact the authors by phone or e-mail were unsuccessful. Blinding did not apply to these cited studies or to withdrawals, follow-up, or intention-to-treat analysis. Study protocols were not available for any included studies. Therefore, it was not possible to evaluate whether all expected outcomes were reported. As such, all the studies were considered as high risk. Zhu et al.'s study [34] was considered as high risk for selection bias in that the admission number was also the randomization method. All the included studies were graded according to the Cochrane Handbook 5.0. Figures 2 and 3 show the authors' judgment of the quality of each methodological parameter.

### 3.3. Effects of the Intervention

#### 3.3.1. Tumor Response

**Figure 4:**
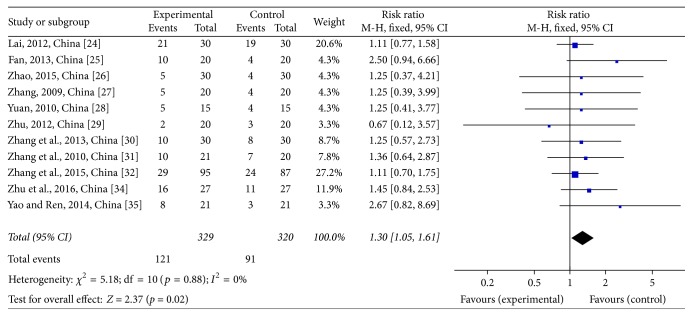
Forest plot of the risk ratio (RR) for the tumor response associated with advanced CRC patients. Notes: the box represents the OR/RR/WMD point estimate of each study, and its area is proportional to the weight of the estimate. Horizontal lines represent the 95% confidence interval (CI).

As shown in Figure 4, there was no significant effect on complete response (CR) or partial response (PR) within the CTJ group (risk ratio (RR) = 1.30; 95% CI: 1.05~1.61; *p* = 0.02; 11 studies [24–32, 34, 35], 649 patients). In these studies, there was no statistically significant effect of JPJD herbs on advanced CRC.

#### 3.3.2. Life Status

**Figure 5:**
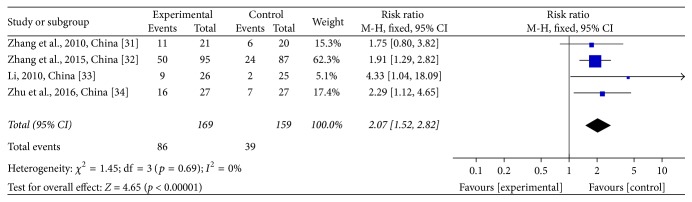
Forest plot of the risk ratio (RR) for the improvement of Karnofsky performance score (KPS) associated with advanced CRC patients. Notes: the box represents the OR/RR/WMD point estimate of each study, and its area is proportional to the weight of the estimate. Horizontal lines represent the 95% confidence interval (CI).

Two types of Karnofsky performance score (KPS) data were reported in the studies: improvements in KPS and KPS data before and after treatment. Only four [31–34] of the 12 studies mentioned an improvement in KPS. For the CTJ group (*n* = 169), 50.89% of patients reported improved KPS data. For the chemotherapy group (*n* = 159), 24.53% of the patients reported improved KPS data. Thus, there was a significant improvement in the CTJ group (RR = 2.07; 95% CI: 1.52~2.82; *p* < 0.00001; four studies, 328 patients) (see Figure 5). There was no heterogeneity among these studies (*χ*^2^ = 1.45, *p* = 0.69, and *I*^2^ = 0%).

**Figure 6:**
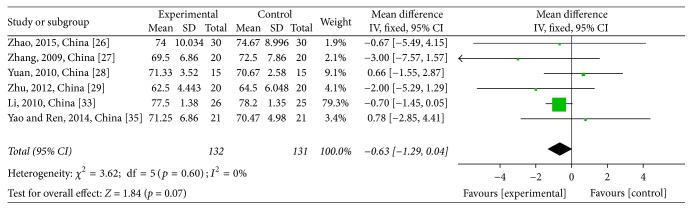
Forest plot of the weighted mean differences (WMD) for the Karnofsky performance score (KPS) of pretreatment associated with advanced CRC patients. Notes: the box represents the OR/RR/WMD point estimate of each study, and its area is proportional to the weight of the estimate. Horizontal lines represent the 95% confidence interval (CI).

**Figure 7:**
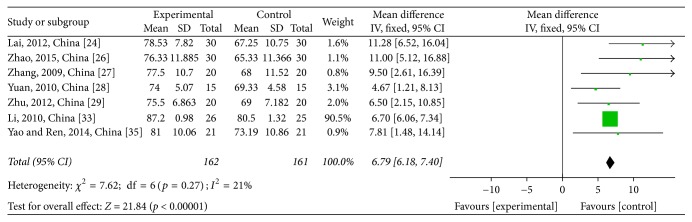
Forest plot of the weighted mean differences (WMD) for the Karnofsky performance score (KPS) of posttreatment associated with advanced CRC patients. Notes: the box represents the OR/RR/WMD point estimate of each study, and its area is proportional to the weight of the estimate. Horizontal lines represent the 95% confidence interval (CI).

Pretreatment KPS data were reported for six studies [26–29, 33, 35] and posttreatment KPS data were reported for seven studies [24, 26–29, 33, 35]. Pretreatment KPS data were not significantly different between the CTJ and chemotherapy groups (WMD = −0,63; 95% CI: −1.29~0.04; *p* = 0.07; *I*^2^ = 0%) (see Figure 6). Heterogeneity among the seven studies was low (*χ*^2^ = 7.62, *p* = 0.27, and *I*^2^ = 21%) and a significant improvement in the CTJ group after treatment was observed (WMD = 6.79; 95% CI: 6.18~7.40; *p* < 0.00001; *I*^2^ = 21%) (see Figure 7).

#### 3.3.3. CM Symptoms' Score and Therapeutic Effect on CM Symptoms

**Figure 8:**
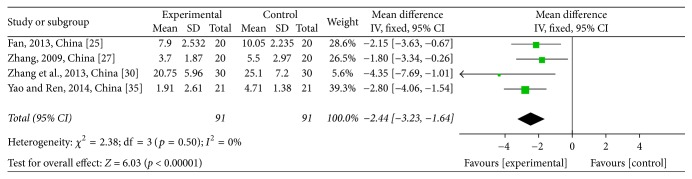
Forest plot of the weighted mean differences (WMD) for Chinese medicine (CM) symptoms' score associated with advanced CRC patients. Notes: the box represents the OR/RR/WMD point estimate of each study, and its area is proportional to the weight of the estimate. Horizontal lines represent the 95% confidence interval (CI).

**Figure 9:**
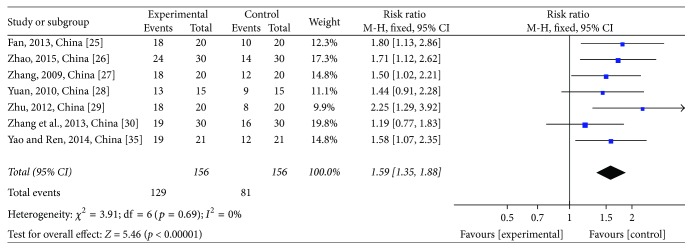
Forest plot of the risk ratio (RR) for therapeutic effect on Chinese medicine (CM) symptoms associated with advanced CRC patients. Notes: the box represents the OR/RR/WMD point estimate of each study, and its area is proportional to the weight of the estimate. Horizontal lines represent the 95% confidence interval (CI).

For analysis of CM symptoms' scores, four studies [25, 27, 30, 35] comprising 182 patients were assessed, with 91 patients in the treatment group and 91 patients in the control group. The heterogeneity test showed *χ*^2^ = 2.38, *p* = 0.50, and *I*^2^ = 0% (see Figure 8). The pooled studies showed a significant decline in the CTJ group (WMD = −2.44; 95% CI: −3.23~−1.64; *p* < 0.00001; *I*^2^ = 0%). Seven studies [25, 27–30, 33, 35] mentioned a therapeutic effect on CM symptoms. The heterogeneity test showed no heterogeneity (*χ*^2^ = 3.91, *p* = 0.69, and *I*^2^ = 0%) (see Figure 9). The pooled studies showed a significant rise in the CTJ group (RR = 1.59; 95% CI: 1.35~1.88; *p* < 0.00001; *I*^2^ = 0%). As shown in the two pooled studies, JPJD herbs improved CM symptoms of advanced CRC patients treated with chemotherapy, although the effect was moderate.

#### 3.3.4. Adverse Events (AEs)

**Figure 10:**
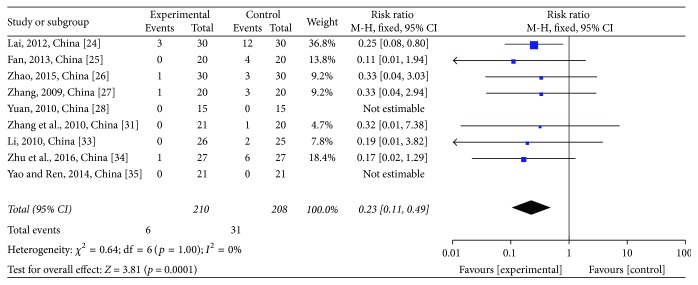
Forest plot of the risk ratio (RR) for nausea and vomiting (N/V) at grades III-IV associated with advanced CRC patients. Notes: the box represents the OR/RR/WMD point estimate of each study, and its area is proportional to the weight of the estimate. Horizontal lines represent the 95% confidence interval (CI).

For the evaluation of nausea and vomiting (N/V) at grades III-IV, nine studies [24–28, 31, 33–35] comprising 418 patients were assessed. Of those patients, 210 were in the treatment group and 208 were in the control group. The heterogeneity test showed *χ*^2^ = 0.64, *p* = 1.0, and *I*^2^ = 0% (see Figure 10). Due to the homogeneity of studies, RR was determined by a fixed-effects model (RR = 0.23; 95% CI: 0.11~0.49; *p* = 0.0001). JPJD herbs reduced the incidence of N/V induced by chemotherapy, although the effect was moderate.

**Figure 11:**
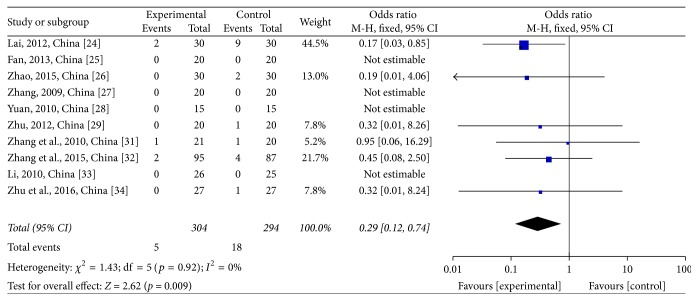
Forest plot of the odds ratio (OR) for platelets (PLT) reductions at the toxicity grades III-IV associated with advanced CRC patients. Notes: the box represents the OR/RR/WMD point estimate of each study, and its area is proportional to the weight of the estimate. Horizontal lines represent the 95% confidence interval (CI).

**Figure 12:**
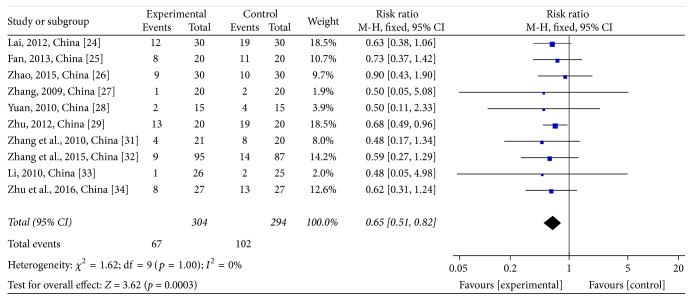
Forest plot of the risk ratio (RR) for platelets (PLT) reductions at the toxicity grades I–IV associated with advanced CRC patients. Notes: the box represents the OR/RR/WMD point estimate of each study, and its area is proportional to the weight of the estimate. Horizontal lines represent the 95% confidence interval (CI).

Two platelet (PLT) stages were reported in these trials. PLT reductions at toxicity grades III-IV were assessed in 10 studies [24–29, 31–34] comprising 598 patients, of which 304 were in the treatment group and 294 were in the control group. However, four [25, 27, 28, 33] of the studies did not reach this stage. The heterogeneity test showed *χ*^2^ = 1.43, *p* = 0.92, and *I*^2^ = 0% (see Figure 11). Due to the homogeneity of the studies, OR was determined by a fixed-effects model (OR = 0.29; 95% CI: 0.12~0.74; *p* = 0.009). The same 10 trials were used to evaluate PLT reductions at toxicity grades I–IV. The heterogeneity test showed *χ*^2^ = 1.62, *p* = 1.0, and *I*^2^ = 0% (see Figure 12). Due to the homogeneity of the studies, RR was determined by a fixed-effects model (RR = 0.65; 95% CI: 0.51~0.82; *p* = 0.0003). JPJD herbs improved PLT values reduced by chemotherapy, although the effect was moderate.

**Figure 13:**
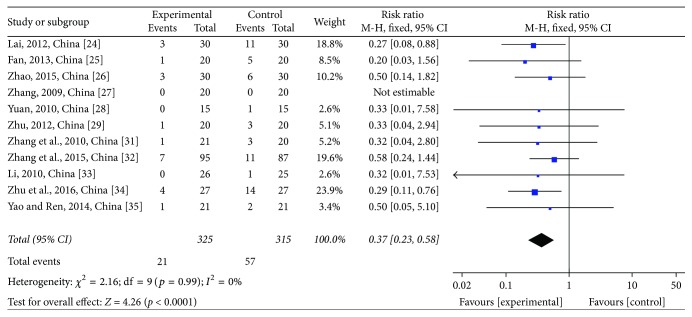
Forest plot of the risk ratio (RR) for the white blood cell (WBC) reductions at the toxicity grades III-IV associated with advanced CRC patients. Notes: the box represents the OR/RR/WMD point estimate of each study, and its area is proportional to the weight of the estimate. Horizontal lines represent the 95% confidence interval (CI).

**Figure 14:**
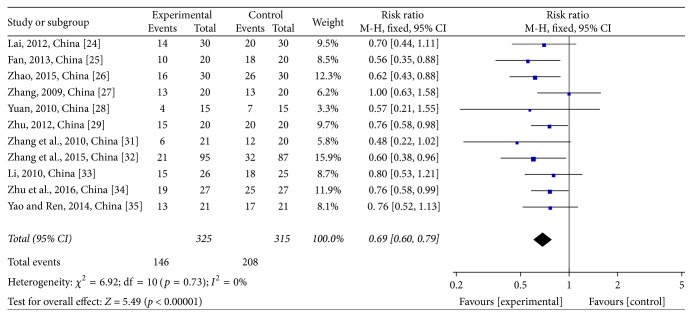
Forest plot of the risk ratio (RR) for the white blood cell (WBC) reductions at the toxicity grades I–IV associated with advanced CRC patients. Notes: the box represents the OR/RR/WMD point estimate of each study, and its area is proportional to the weight of the estimate. Horizontal lines represent the 95% confidence interval (CI).

Two stages of white blood cell (WBC) count were reported in these trials. For WBC reductions at toxicity grades III-IV, 11 studies [24–29, 31–35] comprising 640 patients were assessed, of which 325 were in the treatment group and 315 were in the control group. One study had no patients at this stage. The heterogeneity test showed *χ*^2^ = 2.16, *p* = 0.99, and *I*^2^ = 0% (see Figure 13). Due to the homogeneity of the studies, RR was determined by a fixed-effects model (RR = 0.37; 95% CI: 0.23~0.58; *p* < 0.0001). The same 11 trials were used to evaluate WBC at toxicity grades I–IV. The heterogeneity test showed *χ*^2^ = 6.92, *p* = 0.73, and *I*^2^ = 0% (see Figure 14). Due to the homogeneity of the studies, RR was determined by a fixed-effects model (RR = 0.69; 95% CI: 0.60~0.79; *p* < 0.00001). JPJD herbs improved the WBC that was reduced by chemotherapy, although the effect was moderate.

## 4. Discussion

In recent years, multidisciplinary teams (MDTs) have made significant improvements in evidence-based decision-making [36], which have improved and controlled the condition of advanced CRC patients. TCM may enhance the efficacy of both chemotherapy and radiotherapy [37]. In China, TCM is an important component of the combined therapy of CRC, which results in a reduction in the toxicity associated with chemotherapy [38]. As such, TCM should be part of MDT, allowing for better decision-making.

Chemotherapy is one of the main therapies for treatment of advanced CRC. Combination therapies of fluorouracil with leucovorin and either irinotecan (FOLFIRI regimen [39]) or oxaliplatin (mFOLFOX6 regimen [40]) have become the main CRC chemotherapeutic treatment options. Studies [41, 42] have shown that postoperative CRC patients treated with chemotherapy exhibit bone marrow suppression, gastrointestinal reactions, and other adverse events. In addition, Chinese medicine (CM) experts [43] believe that bone marrow suppression may be caused by spleen deficiency and toxic heat. Invigorating the spleen and detoxification may be key to treating advanced CRC. Hence, this meta-analysis was performed to provide evidence for the use of herbs in advanced CRC patients receiving chemotherapy, that is, Jianpi Jiedu (JPJD), a TCM principle.

In the evaluated trials, prescriptions for JPJD herbs were* Astragalus, Codonopsis, Oldenlandia diffusa, Atractylodes, Poria cocos, and Radix Actinidiae*. These herbs can improve the immune system, alleviate adverse events, improve quality of life, or even be a potential cure. For example, macrocephala polysaccharide is refined from* Atractylodes*. Mao et al. [44] and Tang [45] have suggested that macrocephala polysaccharide of* Atractylodes* can comprehensively enhance the immunity of mice. Tseng et al. [46] found that an* Astragalus membranaceus* extract could inhibit the growth of CRC* in vivo* without apparent toxicity or side effects. Lu et al. [47] found* Oldenlandia diffusa* extracts to inhibit CRC cells* in vitro* and* in vivo*, possibly via activation of AMP-activated protein kinase- (AMPK-) dependent signaling. Kim et al. [48] suggested that an ethanol extract of* Oldenlandia diffusa* may be an effective chemotherapeutic agent for the treatment of human CRC.

This meta-analysis demonstrated JPJD herbs to result in a significant improvement in KPS, a significant improvement in posttreatment KPS (*p* < 0.00001), a significant decline in CM symptoms' score, and a significant rise in the therapeutic effect on CM symptoms (*p* < 0.00001). These results demonstrate the ability of JPJD herbs to alleviate CM symptoms and to improve the KPS of advanced CRC patients undergoing chemotherapy.

Adverse events improved by JPJD herbs were as follows: (1) nausea and vomiting (N/V) at toxicity grades III-IV, (2) reduced PLT numbers at toxicity grades III-IV, (3) decreased PLT numbers at toxicity grades I–IV, (4) decreased WBC at toxicity grades III-IV, (5) and decreased WBC at toxicity grades I–IV. These effects were significant when compared to the control group, which indicates that JPJD herbs reduce the incidence rate of AEs. Analysis of the tumor response to JPJD herbs is ongoing, but no statistically significant effect has been observed to date (*p* = 0.02).

There are many quality of life (QOL) questionnaires, although the European Organization for Research and Treatment of Cancer (EORTC) QLQ C-30 or CR38 is often used. However, the onset, characteristics, and interventions for CRC in China are significantly different from those in Europe and the United States [49]. Use of the KPS scale for QOL evaluation is most commonly used by domestic researchers. However, the KPS scale considers only a portion of QOL and does not fully reflect all characteristics of CM. It does not include the patient's subjective feelings, psychological status, or social status and as such is not comprehensive. In order to accurately assess symptom differences before and after treatment, the CM symptoms' score and the therapeutic effect on CM symptoms were evaluated. The standard table used to score CM symptoms for CRC includes abdominal pain, diarrhea, constipation, anorexia, fatigue, weight loss, nausea, and vomiting. The standard table used to score the therapeutic effect of CM symptoms for CRC includes sallow complexion, fatigue, inappetence, abdominal pain, dry stool, thin sloppy stool, and hemafecia. The two tables differ and either one of them, or both, is chosen by Chinese medicine experts and Chinese medicine researchers. The effects of JPJD herbs on individual symptoms are evaluated below in greater depth.

The anticancer effect of chemotherapy on CRC is the inhibition of DNA synthesis as well as inhibition of the replication of cancer cells [50]. Resultant adverse events include marrow suppression, gastrointestinal reactions, and neurotoxicity. According to “Acute and Sub-acute Reaction Standards for Antineoplastic Agents” from the World Health Organization (WHO), nausea and vomiting, two stages of PLT, and WBC are the major indices of adverse events. This meta-analysis found JPJD herbs to impact bone marrow suppression and the incidence of nausea and vomiting caused by chemotherapy.

As for the tumor response, the reasons why the outcomes are negative may be intrinsic to culture and to system. Differences between traditional Chinese medicine and Western medicine may explain why no tumor response was observed with JPJD herbs. The therapeutic effect of traditional Chinese medicine on tumors is mainly through regulation of the body's internal environment. The purpose is to bring Pi and blood, Yin and Yang, and Zang and Fu into harmony, with the goal of tumor inhibition. Solid tumor parameters are the measures used in Western medicine to assess the tumor response, which is in line with international standards. However, this measurement fails to reflect the approach of traditional Chinese medicine. Some researchers use standards of their own, which may reduce credibility and may be unreliable. In the future, it will be important to design effective and reliable standards that can be applied to traditional Chinese medicine.

The literature related to the use of JPJD herbs in patients with advanced CRC was collected through manual and electronic databases. A formal data extraction table was designed to extract data for quantitative analysis. The search process, the accuracy of the data extraction, and quality assessment were undertaken by two reviewers. Disagreements between the two reviewers were resolved by discussion with a third reviewer. However, this approach does have several limitations and shortcomings. First, the search strategy was restricted to articles published in English or Chinese language, and 12 studies were published exclusively in China. Hence, publication bias may exist. Second, the trial samples sizes varied from 40 to 60 patients (only one trial included 182 subjects) and as such were quite small, with results inconsistent among the trials. Therefore, there may be reporting bias. Third, there may have been information bias in that only limited information was disclosed and only four of the trials used randomization of subjects. None of the selected trials reported allocation concealment or the blinding process. Attempts to contact the authors for more information by phone or e-mail were unsuccessful. Hence, there was a high risk for selection and performance bias in the studies covered by this review. Fourth, only four studies reported improvement in KPS, and only four studies reported the CM symptoms' score. Ten studies reported effects on PLTs, but four of these did not include patient characteristics. No trial reported measures of mortality. All trials reported AEs in the trial and control groups. Selective reporting of outcomes limited an integrated analysis, as did the low quality scores of the included trials.

## 5. Conclusion

This meta-analysis demonstrates the capability of JPJD herbs, when combined with chemotherapy, to increase KPS, increase the therapeutic effect on CM symptoms, decrease the CM symptoms' score, and decrease the rate of AEs. However, due to the complex nature of Chinese herbal medicine (CHM) interventions, further evidence needs to be obtained from high-quality trials with larger sample sizes.

## Figures and Tables

**Table 1:** Characteristics of 12 included trials.

Study ID	Age (yrs)	Sample size (T/C)	Gender (M/F)	Study design (E/C)	Intervention		Treatment session	Main outcomes
					Control group	Experiment group		
Lai, 2012, China [24]	30–73	30/30	38/22	Parallel-group	FOLFOX4	FOLFOX4 + CHM	8 weeks	Tumor response, AEs, KPS
Fan, 2013, China [25]	48–73	20/20	22/48	Parallel-group	L-OHP + S-1	L-OHP + S-1 + CHM	3 weeks	Tumor response, AEs, KPS, CM symptoms score, therapeutic effect of CM symptoms
Zhao, 2015, China [26]	48–67	30/30	39/21	Parallel-group	FOLFIRI	FOLFIRI + CHM	6 weeks	AEs, KPS, therapeutic effect of CM symptoms, QLQ-C30, serum CEA and CA199
Zhang, 2009, China [27]	44–70	20/20	22/18	Parallel-group	OLF	OLF + CHM	6 weeks	Tumor response, AEs, KPS, therapeutic effect of CM symptoms, FACT-C, serum CEA
Yuan, 2010, China [28]	48–71	15/15	12/18	Parallel-group	FOLFOX	FOLFOX + CHM	8 weeks	Tumor response, AEs, KPS, therapeutic effect of CM symptoms, QLQ-C30
Zhu, 2012, China [29]	49–71	20/20	32/08	Parallel-group	FOLFIRI	FOLFIRI + CHM	6 weeks	Tumor response, AEs, KPS, therapeutic effect of CM symptoms, serum CEA
Zhang et al., 2013, China [30]	50–78	30/30	31/29	Parallel-group	Capecitabine	Capecitabine + CHM	6 weeks	Tumor response, CM symptoms score, therapeutic effect of CM symptoms, AEs, EOTRC-QLQ-C30
Zhang et al., 2010, China [31]	NA	21/20	NA	Parallel-group	FOLFOX4	FOLFOX4 + CHM	4 weeks	Tumor response, AEs, KPS
Zhang et al., 2015, China [32]	NA	95/87	105/77	Parallel-group	FOLFIRI/XELOX/ FOLFOX4/FOLFOX6	FOLFIRI/XELOX/FOLFOX4/ FOLFOX6 + CHM	NA	Tumor response, AEs, KPS
Li, 2010, China [33]	25–70	26/25	14/37	Parallel-group	OLF	OLF + CHM	6 weeks	AEs, KPS, FACT-C, QOL, serum CEA and CA199
Zhu et al., 2016, China [34]	34–73	27/27	36/18	Parallel-group	mFOLFOX6	mFOLFOX6 + CHM	8 weeks	Tumor response, AEs, KPS
Yao and Ren, 2014, China [35]	47–72	21/21	22/20	Parallel-group	XELOX	XELOX + CHM	6 weeks	Tumor response, KPS, CM symptoms score, therapeutic effect of CM symptoms

*Notes*. E/C: experiment group/control group; M/F: male/female; CHM: Chinese herbal medicine; N/V: nausea and vomiting; NA: not available; AEs: adverse events; QOL: quality of life; FACT-P: functional assessment of cancer therapy-prostate; QLQ-C: quality of life questionnaire-cancer; KPS: Karnofsky performance score.

## References

[1] Dai Z., Zheng R. S., Zou X. N. (2012). Analysis and prediction of colorectal cancer incidence trend in China. *Zhonghua Yu Fang Yi Xue Za Zhi*.

[2] Soerjomataram I., Lortet-Tieulent J., Parkin D. M. (2012). Global burden of cancer in 2008: a systematic analysis of disability-adjusted life-years in 12 world regions. *The Lancet*.

[3] International Agency for Research on Cancer (IARC) Colorectal Cancer Estimated Incidence, Mortality and Prevalence Worldwide in 2012.

[4] Leake I. (2014). Colorectal cancer: Understanding the routes of metastasis in colorectal cancer. *Nature Reviews Gastroenterology & Hepatology*.

[5] Hubbard J. M., Grothey A. (2015). Colorectal cancer in 2014: Progress in defining first-line and maintenance therapies. *Nature Reviews Clinical Oncology*.

[6] Docherty J. G., McGregor J. R., O'Dwyer P. J., Galloway D. J. (1994). Local recurrence of colorectal cancer: The problem, mechanisms, management and adjuvant therapy. *British Journal of Surgery*.

[7] Tian T., Chen C., Yang F. (2017). Establishment of apoptotic regulatory network for genetic markers of colorectal cancer and optimal selection of traditional Chinese medicine target. *Saudi Journal of Biological Sciences*.

[8] Shi H. Y., Xu Z. F. (2012). Research progression of chemotherapy to colorectal cancer. *Chinese Journal of Cancer Prevention and Treatment*.

[9] Tournigand C., André T., Achille E. (2004). FOLFIRI followed by FOLFOX6 or the reverse sequence in advanced colorectal cancer: a randomized GERCOR study. *Journal of Clinical Oncology*.

[10] Chan S. K., Griffith O. L., Tai I. T., Jones S. J. M. (2008). Meta-analysis of colorectal cancer gene expression profiling studies identifies consistently reported candidate biomarkers. *Cancer Epidemiology, Biomarkers & Prevention*.

[11] O'Connell J. B., Maggard M. A., Ko C. Y. (2004). Colon cancer survival rates with the new American Joint Committee on Cancer sixth edition staging. *Journal of the National Cancer Institute*.

[12] Yin L. L., Jiang C. Y. (2013). Observation on the influence of Shenqi-Fuzheng injection on T-lymphocyte subsets, NK cell and the leukocyte of the patients with advanced gastric cancer. *International Journal of Traditional Chinese Medicine*.

[13] Li X. L., Ma J. (2013). Clinical research progress of combined traditional Chinese and western medicine treatment in treating colorectal cancer. *Modern Journal of Integrated Traditional Chinese and Western Medicine*.

[14] Qin W. F., He J. Q. (2011). Clinical observation of Yiqi Huoxue decotion combined with chemotherapy treating postoperative colorectal cancer patients. *Liaoning Journal of Traditional Chinese Medicine*.

[15] Deng S., Hu B., An H. (2012). Traditional chinese medicinal syndromes and treatment in colorectal cancer. *Journal of Cancer Therapy*.

[16] Wang X. N., Huo J. G. (2007). Explore the thought and method of traditional Chinese medicine treating colorectal cancer. *Journal of Basic Chinese Medicine*.

[17] Ding J. F., Huang Y. S., Li M. H. (2007). Professor Shi Zhiming's clinical experience in treating carcinoma of large intestine. *Shanghai Journal of Traditional Chinese Medicine*.

[18] Chinese Anti-Cancer Association (1999). *New Standard of Diagnosis and Treatment of Common Malignant Tumors*.

[19] Zheng X. Y. (2002). *Clinical Guideline of New Drugs for Traditional Chinese Medicine*.

[20] Internal Medicine of Traditional Chinese Medicine", Planned Textbook for TCM Colleges and Universities in New Century, 2007

[21] Higgins J., Green S. (2010). *Cochrane Handbook for Systematic Reviews of Interventions*.

[22] Jadad A. R., Moore R. A., Carroll D. (1996). Assessing the quality of reports of randomized clinical trials: Is blinding necessary?. *Controlled Clinical Trials*.

[23] Moher D., Pham B., Jones A. (1998). Does quality of reports of randomised trials affect estimates of intervention efficacy reported in meta-analyses?. *The Lancet*.

[24] Lai J. C. (2012). Clinical research on method of strengthening spleen and replenishing Qi and detoxicating and removing blood stasis combined with FOLFOX4 chemotherapy treating advanced colorectal carcinoma. *Liaoning Journal of Traditional Chinese Medicine*.

[25] Fan M. Y. (2013). *The Clinical Study of JianPiBuShenHuaShiJieDu Decotion With L-OHP+S-1 Chemotherapy in the Treatment of Patients With advanced Colorectal Tumor*.

[26] Zhao R. J. (2015). *Clinical research of Tonifying Spleen and Kidney and Dispelling Stasis Detoxification Decoction combined with FOLFIRI chemotherapy in the treatment of advanced colon cancer*.

[27] Zhang Z. Y. (2009). *Clinical study of JianPiHuaShiJieDu Decotion with chemotherapy in the treatment of patients with advanced colorectal tumor*.

[28] Yuan D. (2010). *Theoretical and Clinical Research on Spleen-Tonifying, Dampness-Resolving, Stasis and Toxin-Removing in the Treatment of Advanced Colorectal Carcinoma*.

[29] Zhu L. (2012). *Spleen the Dampness Quyu Detoxification Prescription Combined with FOLFIRI Regimen for Treatment of Advanced Colorectal Cancer*.

[30] Zhang W. W., Chen J., Xie G. Q. (2013). Clinical study of JianPiJieDu decotion combined with capecitabine in the treatment of patients with advanced colorectal tumor. *Journal of Shandong University of Traditional Chinese Medicine*.

[31] Zhang Y., Xu J. H., Sun Y. (2010). Clinical study of Jianpijiedu-Decoction plus FOLFOX4 regimen in the treatment of advanced colorectal cancer. *Global Traditional Chinese Medicine*.

[32] Zhang Y. B., Liu X., Ji Q. (2015). Clinical research on treating metastatic colorectal cancer with Jianpi Jiedu decoction and chemotherapy. *Journal of Chinese Medicine*.

[33] Li P. Y. (2010). *Clinical studies on impact of quality of life of Jianpi Jiedu Huayu therapy in the treatment of patients with advanced colorectal cancer*.

[34] Zhu F. Y., Wang B., Ai Y. (2016). Clinical study of JianPiYiQiJieDu Decotion with chemotherapy in the treatment of patients with advanced colorectal tumor. *Modern Journal of Integrated Traditional Chinese and Western Medicine*.

[35] Yao C., Ren H. C. (2014). Clinical observation of 21 cases advanced colorectal cancer patients with combined traditional Chinese and western medicine treatment. *Journal of New Chinese Medicine*.

[36] Taylor C., Munro A. J., Glynne-Jones R. (2010). Multidisciplinary team working in cancer: what is the evidence?. *BMJ*.

[37] McCulloch M., See C., Shu X. (2006). Astragalus-based Chinese herbs and platinum-based chemotherapy for advanced non-small-cell lung cancer: meta-analysis of randomized trials. *Journal of Clinical Oncology*.

[38] Liu H. R., Liu H. Y., Wang R. C. (2013). Clinical study of Chinese medicine attenuated synergistic effect on colorectal cancer after operation. *Journal of Hebei Traditional Chinese Medicine and Pharmacology*.

[39] Douillard J. Y., Cunningham D., Roth A. D. (2000). Irinotecan combined with fluorouracil compared with fluorouracil alone as first-line treatment for metastatic colorectal cancer: a multicentre randomised trial. *The Lancet*.

[40] De Gramont A., Figer A., Seymour M. (2000). Leucovorin and fluorouracil with or without oxaliplatin as first-line treatment in advanced colorectal cancer. *Journal of Clinical Oncology*.

[41] Chen W. B., Zhu G. J. (2012). Safety observation of FOLFOX regimen in the treatment of advanced colorectal cancer. *Modern Medical*.

[42] Jin X. J., Wang H., Li W. D. (2013). Observation of 33 cases advanced colorectal cancer with FOLFOX6 regimen. *Guide China Medical*.

[43] Zhao J. H., Wei J. L., Zhang T. J. (2013). Effect of Shengyu Decoction on spleen index and tissue pathology of bone marrow depression mice. *Journal of Emergency in Traditional Chinese Medicine*.

[44] Mao J. H., Lv Z. L., Zeng Q. L. (1996). Effects of the polysaccharide of Atractylis macrocephala Koidz on the function of mouse T lymphocyte. *Immunological Journal*.

[45] Tang X. H. (1998). The influence of macrocephala polysaccharide of atractylodes on the immune function of mice. *Traditional Chinese Medicinal Research*.

[46] Tseng A., Yang C.-H., Chen C.-H. (2016). An in vivo molecular response analysis of colorectal cancer treated with Astragalus membranaceus extract. *Oncology Reports*.

[47] Lu P.-H., Chen M.-B., Ji C., Li W.-T., Wei M.-X., Wu M.-H. (2016). Aqueous Oldenlandia diffusa extracts inhibits colorectal cancer cells via activating AMP-activated protein kinase signalings. *Oncotarget *.

[48] Kim B. J., Lee S., Shim J. H., Gim H., Park H. S. (2016). Ethanol extract of oldenlandia diffusa–an effective chemotherapeutic for the treatment of colorectal cancer in humans. *Journal of Pharmacopuncture*.

[49] Wang J. H., Li S. R. (2006). Ten years review of screening and early diagnosis of colorectal cancer in China: 1994–2005. *Gastroenterology*.

[50] Chen W. B., Zhu G. J. (2012). Efficacy and safety of FOLFOX regimen in the treatment of advanced stage colorectal cancer. *Modern Medical (China)*.

